# Exosome-functionalized collagen-coated 3D-printed PCL scaffold for enhanced osteogenic differentiation and bone regeneration: an in vitro and in vivo study

**DOI:** 10.1186/s13036-025-00578-w

**Published:** 2025-11-27

**Authors:** Fatemeh Ghorbani Shemshadsara, Jafar Ai, Mohammad Bayat, Somayeh Ebrahimi-Barough, Sadegh Shirian, Abdolreza Mohamadnia, Mahsa Kouhestani, Moosa Javdani, Mohammad Hossein Karimzadeh, Naghmeh Bahrami

**Affiliations:** 1 Department of Tissue Engineering, School of Advanced Technologies in Medicine, Tehran University of Medical Sciences, Tehran, Iran; 2 Craniomaxillofacial Research Center, Shariati Hospital, Tehran University of Medical Sciences, Tehran, Iran; 3 Department of Biomedical Engineering, University of Minnesota, Minneapolis, MN USA; 4 Department of Pathobiology, Faculty of Veterinary Medicine, Shahrekord University, Shahrekord, Iran; 5 Department of Biotechnology, School of Advanced Technologies in Medicine, Shahid Beheshti University of Medical Sciences, Tehran, Iran; 6 Chronic Respiratory Disease Research Center, NRITLD, Shahid Beheshti University of Medical Science, Tehran, Iran; 7 Department of Applied Cell Sciences, School of Advanced Technologies in Medicine, Tehran University of Medical Sciences, Tehran, Iran

**Keywords:** 3D printed scaffolds, Polycaprolactone (PCL), Collagen-coated scaffolds, Osteoblast-derived exosomes (ODExo), Osteogenic differentiation, Osteogenesis, Bone tissue engineering (BTE)

## Abstract

**Background:**

Bone tissue engineering offers a promising strategy to overcome the limitations of conventional bone grafts, including donor site morbidity and immune rejection. In this study, three-dimensional (3D) polycaprolactone (PCL) scaffolds coated with collagen (Col) were fabricated by extrusion-based printing. The scaffolds were further functionalized with human endometrial mesenchymal stem cells (hEnMSCs) and osteoblast-derived exosomes (ODExo), obtained from osteoblast-like cells, to investigate their potential in bone regeneration.

**Results:**

The printed scaffolds exhibited controlled porosity, suitable mechanical integrity, and improved hydrophilicity following collagen coating. ODExo were isolated from osteoblast-like cells and confirmed by morphology and particle size. In vitro analyses revealed that exosome-functionalized scaffolds significantly enhanced cell adhesion, proliferation, and osteogenic differentiation of hEnMSCs, as indicated by increased calcium deposition and elevated expression of osteogenic markers such as alkaline phosphatase, osteocalcin, RUNX2, and osteonectin, even without osteogenic induction medium. In vivo experiments using a rat calvarial defect model demonstrated superior bone formation and matrix mineralization in scaffolds containing exosomes compared with control groups. Moreover, transplantation of PCL/Col scaffolds seeded with hEnMSCs and ODExo implants further promoted bone regeneration in vivo.

**Conclusions:**

These findings demonstrate that osteoblast-derived exosomes serve as potent bioactive modulators capable of driving osteogenic differentiation and accelerating bone regeneration. Incorporating exosomes into 3D printed PCL/Col scaffolds provides a promising biomimetic and partially cell-free approach for the treatment of critical-sized bone defects. This strategy highlights the potential of combining advanced scaffold fabrication with bioactive vesicles to improve clinical outcomes in regenerative medicine.

**Graphical Abstract:**

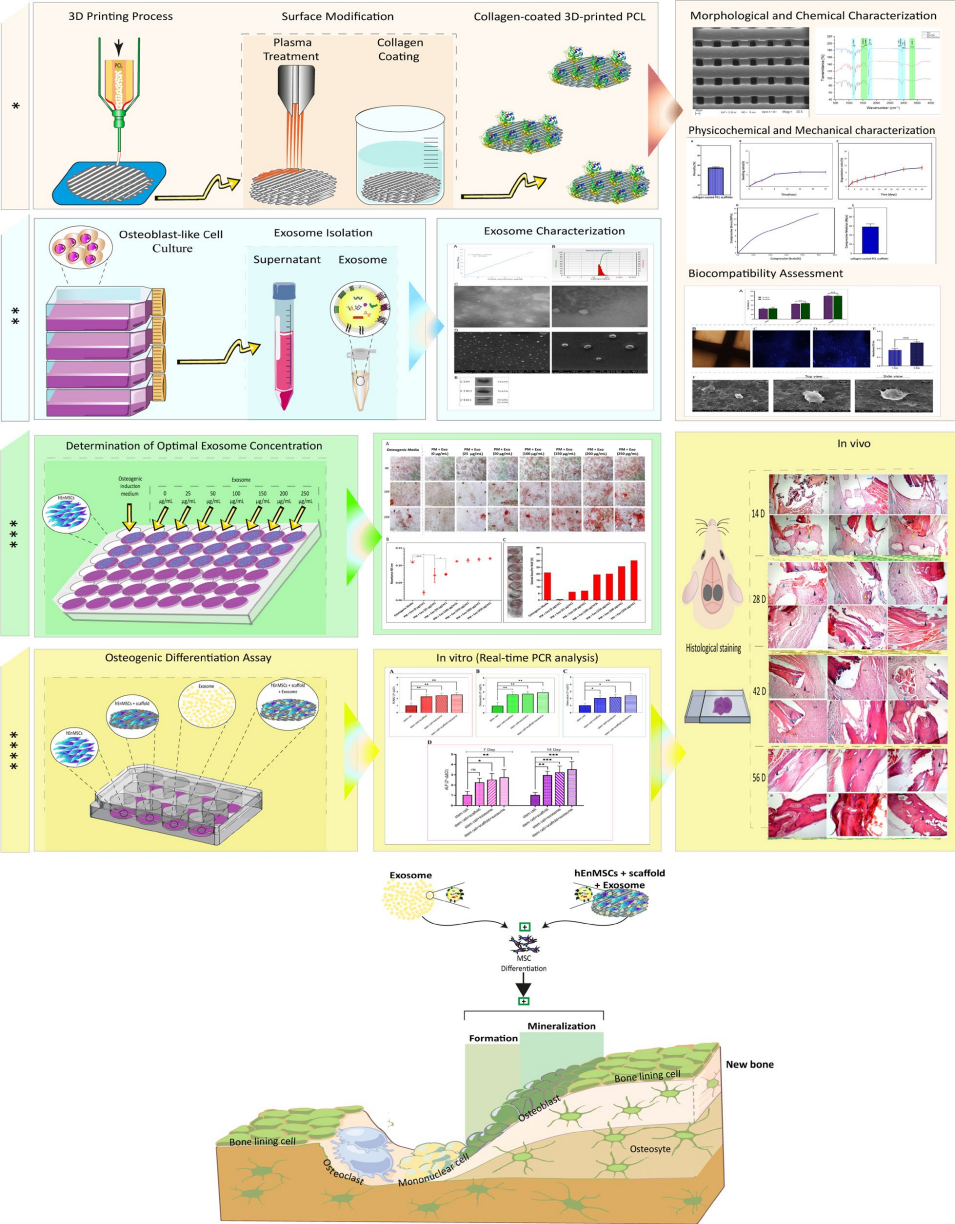

**Supplementary Information:**

The online version contains supplementary material available at 10.1186/s13036-025-00578-w.

## Introduction

The critical role of bone within the skeletal system becomes particularly evident in the context of injuries resulting from trauma, congenital anomalies, tumors, or age-related degeneration [1]. Such conditions often lead to the loss of bone tissue, compromised load-bearing capacity, and diminished protection of internal organs [2].The reconstruction of these defects remains one of the most challenging, costly, and painful procedures in orthopedic surgery. Autologous bone grafts (e.g., iliac crest) and allogeneic transplants have been previously used clinically [3–5]. However, both approaches suffer from significant drawbacks. Autologous grafting is constrained by limited donor availability and carries the risk of additional morbidity at the harvest site due to secondary surgery [6]. Allogeneic grafts are also associated with considerable immunological rejection and high failure rates [7]. To address these limitations, bone tissue engineering (BTE) strategies have been developed, integrating scaffolds, cells, and bioactive cues to enhance osteogenesis, vascularization, and matrix deposition [8–10]. Scaffolds used in BTE with porosity levels exceeding 50% and pore sizes ranging between 200 and 500 micrometers play a pivotal role not only in promoting osteoinduction and osteoconduction but also in serving as effective 3D matrices that facilitate cell adhesion, proliferation, and extracellular matrix (ECM) deposition at the site of injury [6, 11–14]. In recent years, extrusion-based additive manufacturing, known as fused deposition modeling, has become an innovative method for fabricating scaffolds with precise architectural control. This technique enables controlled material deposition, geometric customization, and surface modification, addressing limitations related to tissue heterogeneity and complex design needs. The structural and biological performance of biomimetic scaffolds in bone tissue engineering applications has been significantly enhanced [15–17].

PCL, a linear aliphatic thermoplastic polyester, has garnered significant interest in tissue engineering scaffold fabrication due to its favorable properties, such as biocompatibility, low immunogenicity, cost-effectiveness, ease of processing, and slow degradation rate. PCL is particularly suitable for long-term applications in BTE [18, 19]. PCL’s low melting temperature enhances its compatibility with extrusion-based printing [20]. Bone scaffolds tailored to individual patients, constructed from PCL, provide mechanical support and gradually degrade in vivo, thus obviating the necessity for secondary surgical interventions [21, 22]. The inherent hydrophobicity of native PCL limits cellular adhesion and proliferation. Surface modification strategies, including plasma treatment and subsequent collagen or ECM-protein coating, have been utilized to enhance the hydrophilicity of PCL and improve cell adhesion and bioactivity, thereby supporting BTE [22–24]. Despite advances in bioactive and mechanically supporting scaffolds that mimic the native bone microenvironment, bone regeneration also relies on biological signals derived from cells and their secreted factors for new tissue production.

Among these, mesenchymal stromal cells (MSCs) are non-hematopoietic, multipotent cells with self-renewal capacity, essential for bone regeneration via differentiation into osteogenic lineages and paracrine modulation of the surrounding microenvironment [25–27]. Of the various MSC sources, hEnMSCs have emerged as a significant candidate for BTE platforms. hEnMSCs not only display classical MSC characteristics, including clonogenicity, multipotency, and paracrine activity, but also provide the notable benefit of minimally invasive harvesting. Due to their inherent osteogenic potential and responsiveness to stimuli, hEnMSCs are increasingly utilized as a model for assessing new osteoinductive strategies [28–31].

Beyond the cellular component, bone regeneration is strongly governed by the signaling molecules released into the extracellular environment. Among these, exosomes (Exo) are nanosized extracellular vesicles secreted by various cell types that play a crucial role in mediating intercellular communication [32]. Despite their small size (30–180 nm), they transport a wide range of bioactive molecules, such as proteins, nucleic acids, lipids, and metabolites, which significantly affect intercellular communication and alter the behavior and function of recipient cells [25, 33]. Due to their low immunogenicity, efficient cellular uptake, and extended systemic circulation, exosomes have emerged as promising agents for bone regeneration [34]. These exosomes demonstrate osteoinductive and immunomodulatory properties, making them appropriate for applications as bone tissue engineering scaffolds for repairing critical-sized bone defects [35–37].

While substantial research has focused on the effects of MSC-derived exosomes in bone regeneration, the role of exosomes derived from alternative osteogenic cellular models in promoting osteogenesis has not been fully elucidated. In this study, the ability of osteoblast-derived exosomes to induce osteogenesis within the context of BTE has been investigated. We have developed a 3D-printed porous PCL scaffold coated with collagen (PCL/Col), which, upon incorporation of these vesicles, significantly enhanced the osteogenic differentiation of hEnMSCs in vitro, even in the absence of standard osteogenic medium. Furthermore, in vivo assessments demonstrated improved calvarial bone regeneration. These findings highlight the potential of exosomes-functionalized, cell-free scaffolds as an effective strategy for promoting bone repair in critical-sized defects in a rat model.

## Materials and methods

### Design and fabrication of 3D PCL scaffolds

#### 3D printing process

The fabrication of porous PCL scaffolds was performed using an extrusion-based 3D bioprinter (BioFabX2, Omidafarinan Co., Tehran, Iran) and with Repetier Host V2.1.3 software. The PCL pellets MW = 80,000 g/mol (440744, Sigma-Aldrich) were loaded into a stainless-steel chamber, and the syringe was heated to 110 °C to get a homogeneous polymer melt. The melting PCL was subsequently extruded through a 400 μm nozzle at a pressure of 4.5 bar with a speed of 3 mm/s. The PCL scaffolds were fabricated with a strand width of 500 μm, a pore size of 350 mm, and a layer height of 250 μm, with a layer height of 0.25 mm and an infill density of 55% using a 0°/90° lay-down pattern (Table 1). The scaffolds with different geometries and dimensions were fabricated to meet the specific requirements of each experimental protocol. Disc-shaped scaffolds composed of four layers (5 mm diameter × 1 mm height) were designed for in vitro assessments. For in vivo animal experiments, cylindrical scaffolds with 16 to 20 layers were printed, measuring 5 mm in both diameter and height. Additionally, cubic scaffolds were produced for structural and mechanical characterization. Smaller cubes (5 × 5 × 2 mm; 8–10 layers) were utilized for general scaffold characterization, while larger cubic scaffolds (10 × 10 × 10 mm; 36–40 layers) were specifically fabricated for mechanical testing.

**Table 1:** Parameters for the 3D printing of PCL

Nozzle diameter Extruder temperature Bed temperature Print speed Pressure Layer height Infill density Infill pattern direction	400 μm 110 °C 25 °C 3 mm/s 4.5 bar 0.25 mm 55% 0°/90°

#### Surface modification

Cleaning and sterilization of the scaffolds is the first step in applying the coating. To achieve this, the final 3D-printed PCL scaffolds were sequentially immersed in an ascending ethanol series (30%, 50%, 70%, 80%, 96%, and 100%), with five repetitions at each concentration. Next, the surfaces of dried PCL scaffolds were activated using a low-pressure plasma system (Zepto, Diener Electronic, Germany) to enhance their hydrophilicity and improve collagen coating adhesion of the PCL scaffolds. The process was performed in a reaction chamber at 0.3 mbar with oxygen plasma (O₂) at 50 W. The scaffolds measuring 1 mm and 5 mm in height were treated for 120 s, while those with a height of 10 mm were treated for 3 min.

#### Collagen coating

Type I collagen solution was prepared by employing collagen powder (C7661, rat tail; Sigma-Aldrich) as previously described by Liao, Lee [38]. Briefly, a final volume of 50 mL required for collagen coating the scaffolds was formulated by dissolving 0.25 g of sterilized type I collagen powder in sterile 0.1% v/v acetic acid, resulting in an achievement of collagen concentration of 5 mg/mL. Following plasma treatment, the scaffolds were placed into a 48-well plate, with each well containing 800 µL of the prepared collagen solution. The plate containing both the scaffolds and collagen solution was then incubated on a moving platform at 4 °C for 72 h. After incubation, the scaffolds were removed from the collagen solutions and allowed to dry for 24 h at 37 °C. Subsequently, the dried scaffolds were immersed in a 2/5% glutaraldehyde solution (Merck, Darmstadt, Germany) in water for 15 min inside a sealed chamber to promote membrane bonding under glutaraldehyde treatment. This process enhances the strength and stability of coated collagen. After incubation, the scaffolds underwent five sequential washes with deionized water, each lasting 10 min, before being dried on filter paper.

### Morphological and chemical characterization

#### Morphological analysis

Morphologically, the printed scaffolds (*n* = 3) were characterized using an SEM (Leo 1430 VP, Zeiss, Germany). The samples were sputter-coated with gold for 60 s and imaged at an accelerating voltage of 10 kV. The average pore size and strand diameter were quantified by calibrating the scale bars on the SEM micrographs using Fiji software (ImageJ v1.52 h, National Institutes of Health, Bethesda, MD, USA).

#### Fourier transform infrared spectroscopy (FTIR)

To confirm the presence of collagen coating on the surface of PCL scaffolds, the functional groups and chemical composition of the scaffolds were analyzed using FTIR (FT-IR-4700, JASCO, Japan) over a spectral range of 500 cm⁻¹ to 4000 cm⁻¹.

### Physicochemical and mechanical characterization

#### Porosity measurements

The porosity of the collagen-coated 3D-printed scaffolds was evaluated using the fluid displacement method [19]. Ethanol was selected due to its penetration ability into the scaffold’s pores. The scaffold was submerged in a graduated cylinder filled with ethanol (V1) until it became fully saturated and floated. The total volume of ethanol and the saturated scaffold was recorded as (V2). Subsequently, the scaffold was removed, and the remaining volume of ethanol was measured as (V3). The porosity was then calculated using the following formula:

$$Porosity\:ratio\left(\%\right)=\frac{\left(v_1-v_3\right)}{\left(v_2-v_3\right)}\ast100$$

#### Swelling ratio

Initially, the dry weight of lyophilized PCL scaffolds coated with collagen (dimensions: 10 × 10 × 1 mm; *N* = 3) was measured (Wd). Subsequently, the scaffolds were immersed in 10 mL of phosphate-buffered saline (PBS) and incubated at 37 °C. For predefined intervals (2, 4, 8, 16, and 24 h) during the testing period, the scaffolds were taken out of the solution and meticulously weighed (Ws) after gently blotting away excess surface moisture with filter paper. The swelling ratio of the scaffolds was calculated according to the following equation:

$$\text{Swelling\:ratio}:\frac{\text{Ws}-\text{Wd}}{Wd}\times100$$

#### Degradation rate

Degradation rate were measured to quantify the weight loss of the scaffolds under in vitro conditions. To begin the procedure, the scaffolds were subjected to freeze-drying, and their initial dry weight (W0) was meticulously recorded. Each sample was then immersed in 10 mL of PBS buffer (pH 7.4) and kept at 37 °C (*N* = 3) to mimic physiological conditions. At 1, 3, 7, 14, 21, 30, 45, and 60 days, the scaffolds were removed from the solution, and excess surface moisture was carefully blotted with filter paper. The samples were then precisely weighed to determine their remaining dry weight (Wt). The degradation rate, expressed as a percentage of weight loss, was determined based on the formula presented below:

$$\text{Degradation\:rate}\left(\%\right)=\frac{\text{W}0-\text{Wt}}{W0}\times100$$

#### Mechanical test

Compression tests were performed using a Hounsfield H25KS universal testing machine (Hounsfield H25KS, England) equipped with a 2500 N load cell at room temperature (RT). Cube-shaped scaffolds were compressed at a constant crosshead speed of 1 mm/min up to 50% strain. Stress–strain data were derived from load–displacement curves. The compressive modulus was calculated from the slope of the initial linear portion (up to approximately10% strain) of the stress–strain curve.

### Biocompatibility assessment for 3D printed scaffolds

#### Culture of human endometrial mesenchymal stem cells

Human endometrial MSCs (IBRC C11200) were purchased from the Iranian Biological Resource Center (IBRC, Tehran, Iran). The cells were cultured in proliferation medium, consisting of Dulbecco’s Modified Eagle Medium/Nutrient Mixture F-12 medium (DMEM/F-12) supplemented with 15% fetal bovine serum (FBS) (Gibco, USA) and 1% penicillin-streptomycin (Pen/Strep) (Bio Idea Co., Iran) at 37 °C in a humidified incubator with 5% CO₂. Passages 4 to 6 hEnMSCs were used for all the assays [39].

#### Cell viability assessment

To evaluate cell viability, the hEnMSCs were seeded on collagen-coated 3D-printed PCL scaffolds and on tissue culture polystyrene (TCP) as a control for 5 days. Cell viability was evaluated using the 3-(4,5-dimethylthiazol-2-yl)-2,5-diphenyl tetrazolium bromide (MTT) assay (Sigma, Germany). The collagen-coated scaffolds were initially washed with 70% ethanol, sterilized with ultraviolet (UV) light on each side, and then incubated in a 96-well plate containing culture medium overnight. The hEnMSCs (10⁴ cells per 5 mm scaffold) were seeded onto the scaffolds and TCP under standard conditions (37 °C, 5% CO₂) for 1, 3, and 5 days. After the designated time points, the culture medium was carefully removed, and the cell-containing scaffolds were washed gently with PBS. Subsequently, 100 µL of MTT solution (5 mg/mL in the DMEM) was added to each well (*n* = 5). For conversion of MTT into formazan crystals by mitochondrial dehydrogenases of living cells, the plate was incubated at 37 °C for 3–4 h. To dissolve the dark-blue intracellular formazan crystals, the supernatant was removed and 200 µL of dimethyl sulfoxide (DMSO) was added to each well. Absorbance was measured at 570 nm using an ELISA reader (ICN, Switzerland).

#### DAPI staining

The proliferation and attachment of hEnMSCs on collagen-coated PCL scaffolds were assessed using MTT and DAPI (4′,6-diamidino-2-phenylindole) staining. On days 1 and 3 post-incubation, the culture medium was removed, and the PCL/Col scaffolds were fixed with 4% paraformaldehyde (PFA, Sigma-Aldrich) for 20 min. Following washing with 1× PBS, 500 µL of DAPI staining solution (1 µg/mL, Sigma) was applied to each scaffold under light-protected conditions and incubated for 5 min. The samples were then rinsed with PBS and visualized using a fluorescence microscope (Olympus BX51, Japan).

#### Cell–scaffold interaction

Morphology and adhesion of the cell seeded on the scaffolds were assessed using an SEM. The hEnMSCs were seeded onto each scaffold at the same density as outlined earlier. On day 3 post-incubation, the cell/ PCL/Col scaffold constructs were fixed with 2.5% glutaraldehyde for 2 h, followed by thorough rinsing with PBS. A stepwise ethanol gradient with increasing concentrations was applied to dehydrate the samples at 37 °C for 15 min, and then they were air-dried overnight in a laminar flow hood. The dried samples were sputter-coated with gold at a current of 6 mA, forming an approximately 5 Å gold layer on the surface, and were examined using an FEI ESEM Quanta 200 at an accelerating voltage of 25 kV.

### Extraction and identification of exosomes

For exosome isolation, human osteoblast-like cells (Saos-2) were obtained from the Pasteur Institute of Iran (Tehran, Iran). The cells were seeded in T75 flasks and cultured in DMEM supplemented with 10% FBS and 1% penicillin-streptomycin under a 5% CO₂ atmosphere at 37 °C. Once the cells reached 80% confluence, the culture medium was removed, and the cells were washed three times with PBS before being incubated in a complete culture medium containing 10% exosome-depleted FBS for 48 h. The conditioned medium was then collected, and exosomes were isolated from the flask supernatants using the ExoSun kit (ExoSun, Iran) following the manufacturer’s instructions. Briefly, the collected medium was centrifuged at 3,000 × g for 10 min to remove cellular debris, followed by centrifugation at 8,000 × g for 60 min to eliminate EVs larger than 200 nm. The supernatant was then combined with reagents A and B at an 8:1 ratio and shaken for 16 h. Finally, the resulting pellet was dissolved in 500 µL of reagent C and stored at − 20 °C for further analysis. All centrifugation and shaking procedures were conducted at 4 °C.

The total protein content of isolated exosomes was quantified using the Bradford Protein Assay Kit (DNAbiotech, Iran) to determine their concentration. The size distribution of osteoblast-derived exosomes was evaluated using dynamic light scattering (DLS) with a Nanotrac Wave II (Microtrac, Inc.). Morphological and size characteristics were further analyzed via transmission electron microscopy (TEM; Zeiss, 100 kV) following negative staining with 2% uranyl acetate for 1–2 min. FEI ESEM was also performed to confirm the shape and size of the exosomes. Additionally, characteristic exosomal proteins, including CD9, CD63, and CD81 (1:1000; Santa Cruz Biotechnology, Inc.), were analyzed via western blotting.

### In vitro evaluation of osteogenesis

#### Exosome dose optimization for osteogenesis

To determine the optimal concentration of ODExo for inducing osteogenesis, hEnMSCs were seeded at a density of 1 × 10⁵ cells per well in 48-well culture plates. Upon reaching 70–80% confluency, seven experimental groups were treated with proliferation medium consisting of DMEM/F-12 supplemented with 10% FBS and 1% Pen-Strep, each receiving a different concentration of exosomes (0, 25, 50, 100, 150, 200, and 250 µg/mL) to assess their osteoinductive potential under non-differentiating conditions. A separate group was cultured in osteogenic induction medium containing 100 nM dexamethasone, 10 mM β-glycerophosphate, and 0.2 mM ascorbic acid (all from Sigma-Aldrich, St. Louis, MO, USA), and served as a positive control. All groups were incubated for 14 days at 37 °C in a humidified atmosphere with 5% CO₂. The culture media were refreshed every 3 days.

At the end of the culture period, hEnMSCs osteogenic differentiation was assessed by evaluating mineralized matrix formation (calcium deposition) via Alizarin Red S (ARS) staining. Each well was gently rinsed three times with PBS to eliminate any remaining medium. The cells were subsequently fixed using 500 µL of 4% paraformaldehyde for 15 min at RT. After fixation, a 2% Alizarin Red S solution, adjusted to pH 4.2, was added to the wells and incubated for 15 min under mild shaking conditions. To remove unbound dye, the wells were thoroughly washed with distilled water until the rinsing solution became completely clear, ensuring selective staining of calcium-enriched areas. The stained wells were imaged using an Olympus IX73 microscope with DP Controller software (Olympus Corp., Tokyo, Japan). For quantitative evaluation of calcium deposition, the bound dye was extracted by adding 200 µL of 10% acetic acid, followed by centrifugation. The supernatant (200 µL) was neutralized with 75 µL of 10% ammonium hydroxide, and 100 µL of the neutralized solution was transferred to a 96-well plate. Absorbance was measured at 405 nm using a microplate reader (BioTek ELx808) to quantify mineral content. Additionally, captured images were analyzed in ImageJ by converting to 8-bit, thresholding, and measuring the stained area percentage per well.

#### Gene expression

Quantitative analysis of osteogenesis-related genes in hEnMSCs, including alkaline phosphatase (ALP), osteocalcin (OCN), Runt-related transcription factor 2 (RUNX2), and Osteonectin (ON) was performed using quantitative Real-time PCR(qRT-PCR). Gene expression was analyzed on day 7 post-induction, while the ALP gene was additionally assessed on day 14 to examine its sustained activity during the later stages of osteogenic differentiation. Total RNA was extracted using TrizoLEX reagent (DNAbiotech, Iran) from four experimental groups: untreated hEnMSCs, hEnMSCs seeded on collagen-coated PCL scaffolds, hEnMSCs treated with exosomes, and hEnMSCs seeded on the same scaffolds followed by direct application of exosomes, allowing their penetration into the scaffold pores. Complementary DNA (cDNA) was synthesized from 1 µg of RNA using the Viva 2-step RT-PCR Kit (Cat. No. RTPL12, Vivantis, Malaysia), according to the manufacturer’s protocol.

The qRT-PCR was carried out using a 7300 Real-Time PCR System (Applied Biosystems, USA) and SYBR Green Master Mix (Takara, Japan). Each 20 µL reaction mixture contained 3 µL of cDNA template, 5 µL SYBR Green Master Mix, 2 µL of each primer (forward and reverse), and 10 µL of nuclease-free water. Thermal cycling conditions were as follows: initial denaturation at 95 °C for 30 s, followed by 40 cycles of 95 °C for 20 s, 62 °C for 60 s, and 72 °C for 30 s. All samples were run in triplicate. Cycle threshold (Ct) values were automatically calculated by the system software, and relative gene expression levels were determined using the 2^(-ΔΔCt) method. β-actin served as the internal reference gene. Primer sequences are provided in Table 2.

**Table 2:** Primer sequences used in quantitative real-time PCR targeting osteogenesis-related genes

Osteogenesis marker genes	Primer sequences	
	Forward reverse	
ALP Runx2 Osteocalcin Osteonectin β-actin	GCCTTGCTCACTCACTCACT AATGCCTCTGCTGTTATGAA GCAAAGGTGCAGCCTTTGTG AGGTATCTGTGGGAGCTAATC AGCACAGAGCCTCGCCTT	ACAGGAGAGTCGCTTCAGAGA CTTCTGTCTGTGCCTTCTG GGCTCCCAGCCATTGATACAG ATTGCTGCACACCTTCTC CACGATGGAGGGGAAGAC

### Animal study

Seventy-two adult male Wistar rats (250 ± 20 g) were obtained from a breeding colony. They were kept in individual cages for one week before beginning the experiment at the animal house center of the university according to the international guidelines for animal experimentation. The rats were allowed to have free access to a standard laboratory diet and tap water by the end of the study. For creation of a full-thickness defect on the calvaria bone, the rats were anesthetized by intraperitoneal injection of ketamine (30 mg/kg) and xylazine (10 mg/kg). The skull skins were firstly shaved and prepared aseptically with povidone iodine. A circular critical-sized defect with a diameter of 1 cm was created in the middle part of each calvaria bone using an electrical motor. The rats were injected intramuscularly with 2 mg/kg of gentamicin (2 mg/kg) post-operation [40]. After creating full-thickness critical-size calvarial bone defects, the rats were randomly divided into six groups (12 rats per group): control (bone defect without treatment), Exo, hEnMSCs, PCL/Col, PCL/Col/Exo, and PCL/Col/Exo/hEnMSCs. The full-thickness critical size calvaria bone defects were filled using the synthesized scaffolds. In the Exo and hEnMSC groups, rats received exosomes (0.2 mL, 100 µg) and/or 1 × 10⁵ hEnMSCs immediately after surgery. The isolated exosomes were administered not only directly to the defect site but also via intraperitoneal injection. For scaffold implanted groups (PCL/Col/Exo and PCL/Col/Exo/hEnMSCs), exosomes were applied directly onto the scaffolds at the defect site, allowing penetration into the scaffold pores. On days 14, 28, 42, and 56 post-treatment the bone defects were harvested, and the effective effect of each approach was then analyzed using histopathological investigations.

#### Histopathological evaluation

For histological evaluation of bone regeneration on days 14, 28, 42, and 56 post-treatments, the rats were firstly anesthetized and then sacrificed. Their treated calvarias were removed and free of soft tissues dissected. The collected Calvaria containing the treated defect were fixed in 10% formalin buffer for one week. After fixation, the bone samples were decalcified using EDTA for three days. The samples were processed and stained for histological analysis using hematoxylin and eosin (H&E) staining. For investigation of bone regeneration histologically, 5 μm in thickness paraffin embedded bone tissue sections were prepared from the centers of each bone sample and stained with H&E. The stained sections were blindly analyzed and scored by an expert pathologist based on previous studies [41, 42].

### Statistical analysis

The SPSS software, version 22, was used for analysis of the data. The analyzed data are finally expressed as mean ± standard deviation. Parametric and non-parametric data were analyzed using the one-way ANOVA and Kruskal-Wallis tests, respectively, followed by post hoc Tukey and Mann-Whitney tests. Two-way ANOVA followed by Tukey’s post-hoc test was also used to evaluate the effects of group (control vs. Scaffold) and time (1, 3, and 5 days) on cell viability. An unpaired t-test was used to compare cell proliferation between day 1 and day 3 for both scaffolds *P* < 0.05 was considered statistically significant.

## Results

### Morphological and chemical characterization

**Fig. 1:**
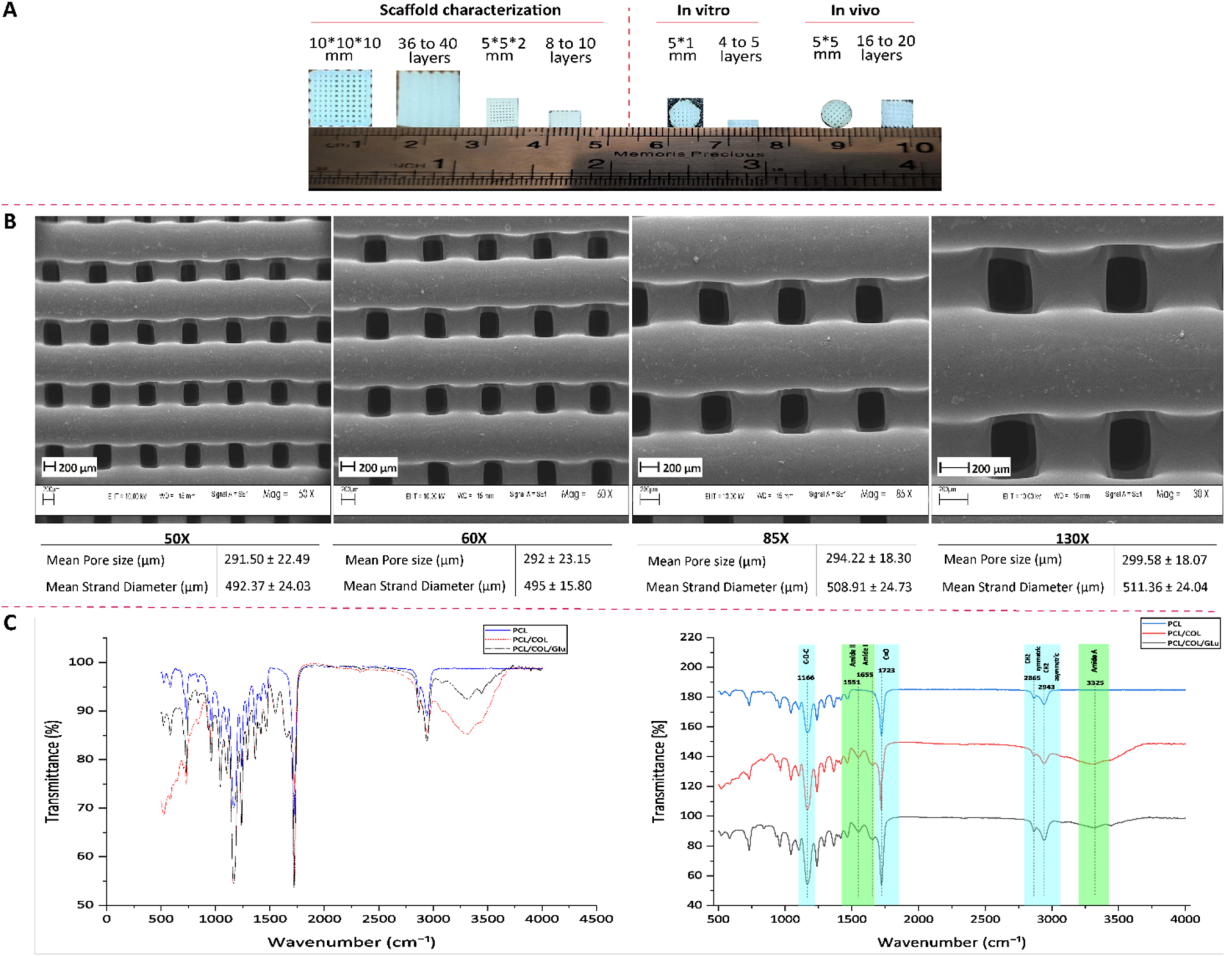
Morphological characterization of 3D-printed PCL scaffolds and chemical analysis of collagen coating: (**A**) Macroscopic view of various 3D-printed PCL scaffolds fabricated for different tests, showing geometry and number of printed layers. (**B**) SEM images at magnifications ranging from 50× to 130×, illustrating scaffold morphology along with measured pore sizes and strand diameters. (**C**) ATR-FTIR spectra of PCL, PCL/COL, and glutaraldehyde-crosslinked PCL/COL scaffolds. The left panel presents the raw spectra, while the right panel highlights characteristic peaks of PCL and collagen, confirming successful surface coating

The overall dimensions and the number of layers of the various 3D-printed PCL scaffolds and morphological details detected by SEM at four magnifications (50×, 60×, 85×, and 130×) are shown in Fig. 1A and B, respectively. For each magnification, the pore size and strand diameter were measured, and the corresponding quantitative values are presented alongside each SEM image for clarity. The average pore size across all magnifications was 294.37 ± 20.50 μm, while the average strand diameter was 502.11 ± 22.15 μm. Although slight variations in measurements were observed among magnifications, the results remained consistent with the expected scaffold design. All samples were fabricated using the same needle gauge and printing parameters to ensure structural uniformity.

The FTIR spectra of PCL, PCL/Col, and PCL/Col crosslinked with glutaraldehyde, aimed at confirming collagen coating on PCL scaffolds are shown in Fig. 1C. The PCL spectrum showed characteristic bands at 2943 cm⁻¹ and 2865 cm⁻¹, attributed to the asymmetric and symmetric stretching vibrations of the CH bonds in CH₂ groups, respectively. Other peaks of PCL at 1723 cm⁻¹ and 1166 cm⁻¹ were observed. The peak at 1723 cm⁻¹ attributed to the stretching vibrations of the carbonyl ester (C = O), while the peak at 1166 cm⁻¹ corresponded to the stretching of the C-O-C groups in PCL. Along with these peaks observed in the PCL scaffolds coated with either uncrosslinked or glutaraldehyde-crosslinked Col, three distinct peaks were also detected, which were associated with Col bands. A peak at 3325 cm⁻¹ was detected in the Col-coated PCL scaffolds, attributed to NH stretching and corresponding to the amide A band of Col. The broadening of this peak is likely due to hydrogen bonding interactions within the Col network. Peaks at 1655 cm⁻¹ and 1551 cm⁻¹ attributed to the stretching vibration of amide I (C = O) and amide II (NH), respectively, were also detected. Based on these results, it can be concluded that the Col layer was successfully coated on the surface of PCL scaffolds.

### Physicochemical and mechanical characterization

**Fig. 2:**
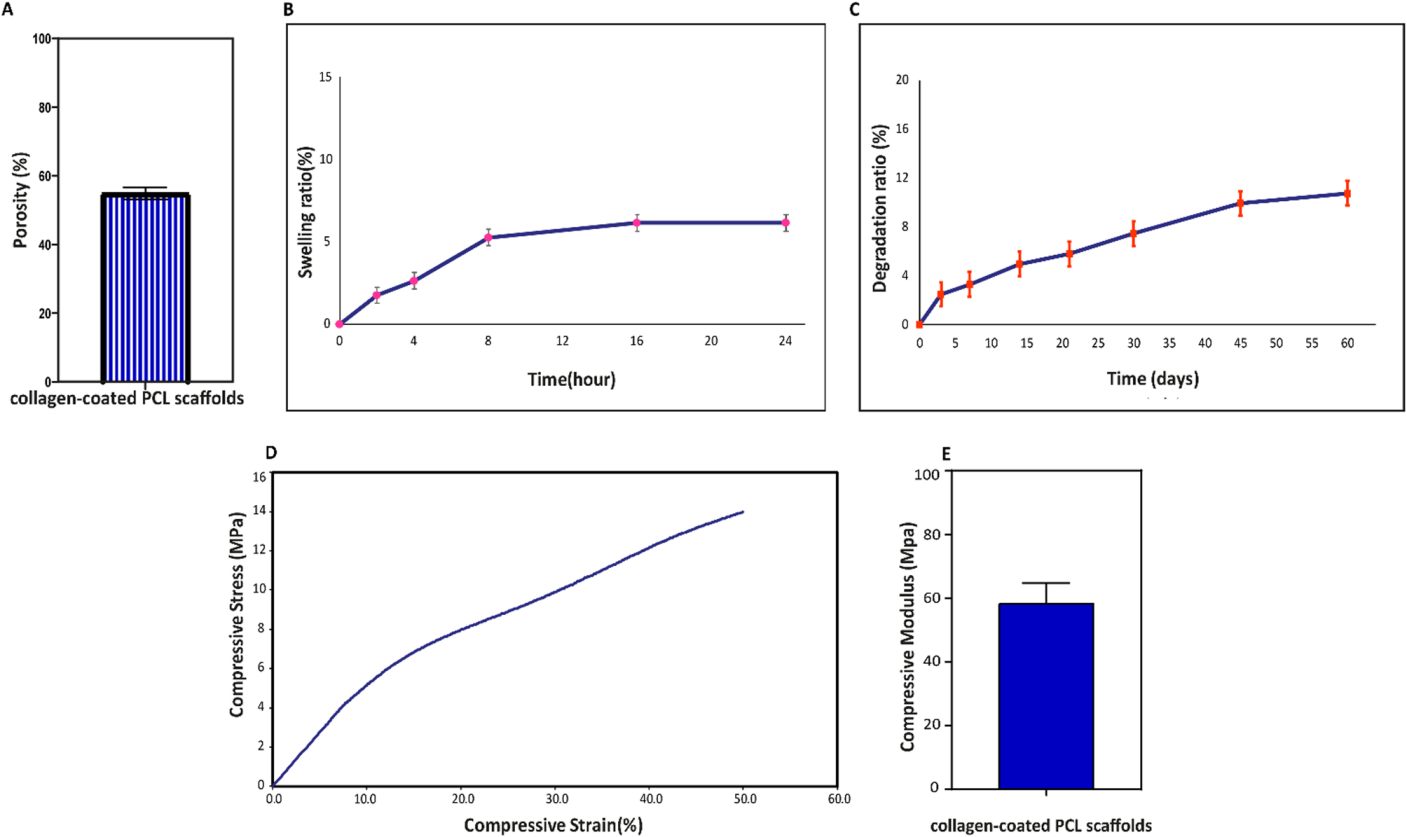
Physicochemical and mechanical characterization of the collagen-coated 3D-printed PCL scaffold: (**A**) Porosity, (**B**) Swelling behavior, (**C**) Degradation profile, (**D**) Compressive stress–strain curve, and (**E**) Compressive modulus of the scaffold

The porosity of the Col-coated 3D-printed scaffolds was found to be 53.11 ± 0.69% (Fig. 2A). The swelling ratio was measured at predefined time points following immersion in PBS at 37 °C. Results showed a marked increase within the first 2 h, which continued progressively at 4 and 8 h. The maximum swelling ratio reached to 6.14 ± 0.5%, after 16 h, highlighting the hydrophilic nature of the Col coating and the scaffold’s porous structure. This swelling behavior remained consistent throughout the 24-hour evaluation period (Fig. 2B).

The gradual degradation profile of the scaffolds as a function of time over a 60-day period is presented in Fig. 2C. Immersion in PBS resulted in a progressive reduction in scaffold weight. The minimum and maximum degradation rates recorded at days 3 and 60 were 2.47 ± 1% and 10.74 ± 1%, respectively, demonstrating a controlled and predictable degradation pattern. The compression stress-strain curves are presented in Fig. 2D. Mechanical testing revealed that the compressive modulus of the Col-coated PCL scaffold was 58.4 ± 6.34 MPa, as depicted in Fig. 2E.

### Biocompatibility assessment for 3D printed scaffolds

**Fig. 3:**
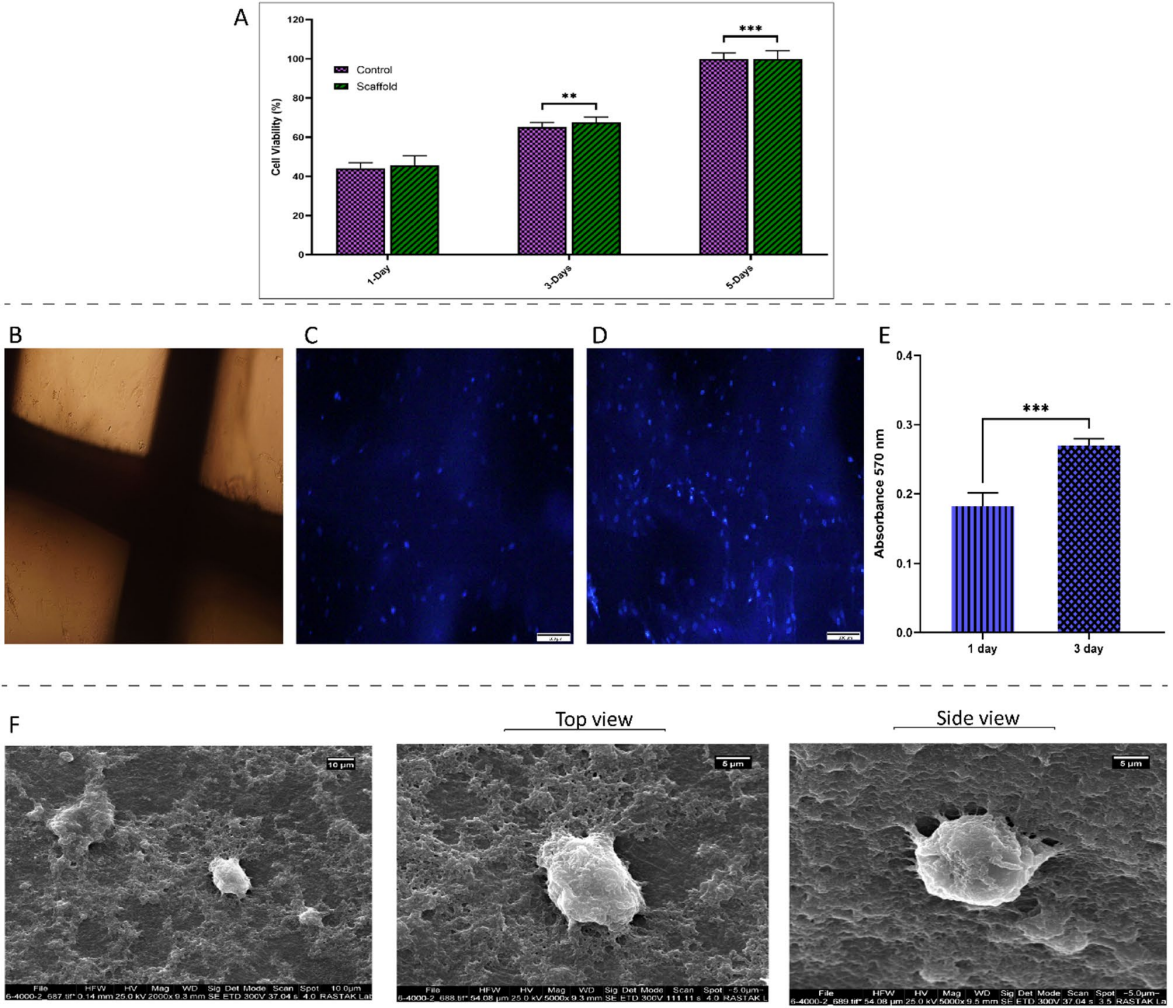
Cell culture tests on collagen-coated 3D-printed PCL scaffolds: (**A**) MTT assay assessing the viability of hEnMSCs cultured on PCL/Col scaffolds at days 1, 3, and 5. (**B**) Morphology of collagen-coated scaffolds prior to DAPI staining. (**C**, **D**) DAPI-stained hEnMSCs on PCL/Col scaffolds at days 1 and 3, respectively. (**E**) Proliferation assay of hEnMSCs at the same time points. (**F**) Scanning Electron Microscopy (SEM) images illustrating cell adhesion on scaffolds after 3 days of culture. Scale bars: 50 μm (DAPI), 10 μm and 5 μm (SEM). Data are presented as mean ± SD (*n* = 3), with statistical significance denoted by **(*p* < 0.01), ***(*p* < 0.001)

The MTT assay was performed to assess the viability of hEnMSCs cultured on collagen-coated 3D-printed PCL scaffolds at days 1, 3, and 5 post-culture. As shown in Fig. 3A, cell viability significantly increased in both the PCL/Col scaffold and TCP control groups on days 3 and 5. These findings confirm that none of the scaffolds negatively affected cell viability, further supporting their biocompatibility.

To further evaluate cell attachment and proliferation, DAPI staining was conducted on the collagen-coated PCL scaffolds. To provide a comparative visualization, the scaffold morphology before DAPI staining is shown in Fig. 3B, while Fig. 3C and D depict the blue-stained cell nuclei at 1 and 3 days after cell seeding, respectively. The examined figures indicate that MSCs show a homogeneous distribution on the scaffolds and that the density of living cells increase over the 3-day culture period. Additionally, the MTT proliferation assay results on the mentioned time points confirm the enhanced cell proliferation (Fig. 3E).

Cell adhesion was evaluated using FEI ESEM images after 3 days. In Fig. 3F, the images depict the cell morphology from both top and side views, providing a comprehensive perspective on cell attachment and spreading on the PCL/Col scaffolds. These findings underscore the suitability of the PCL/Col scaffold as a supportive microenvironment for promoting cell adhesion.

### Characterization of osteoblast-like cell-derived exosomes

**Fig. 4:**
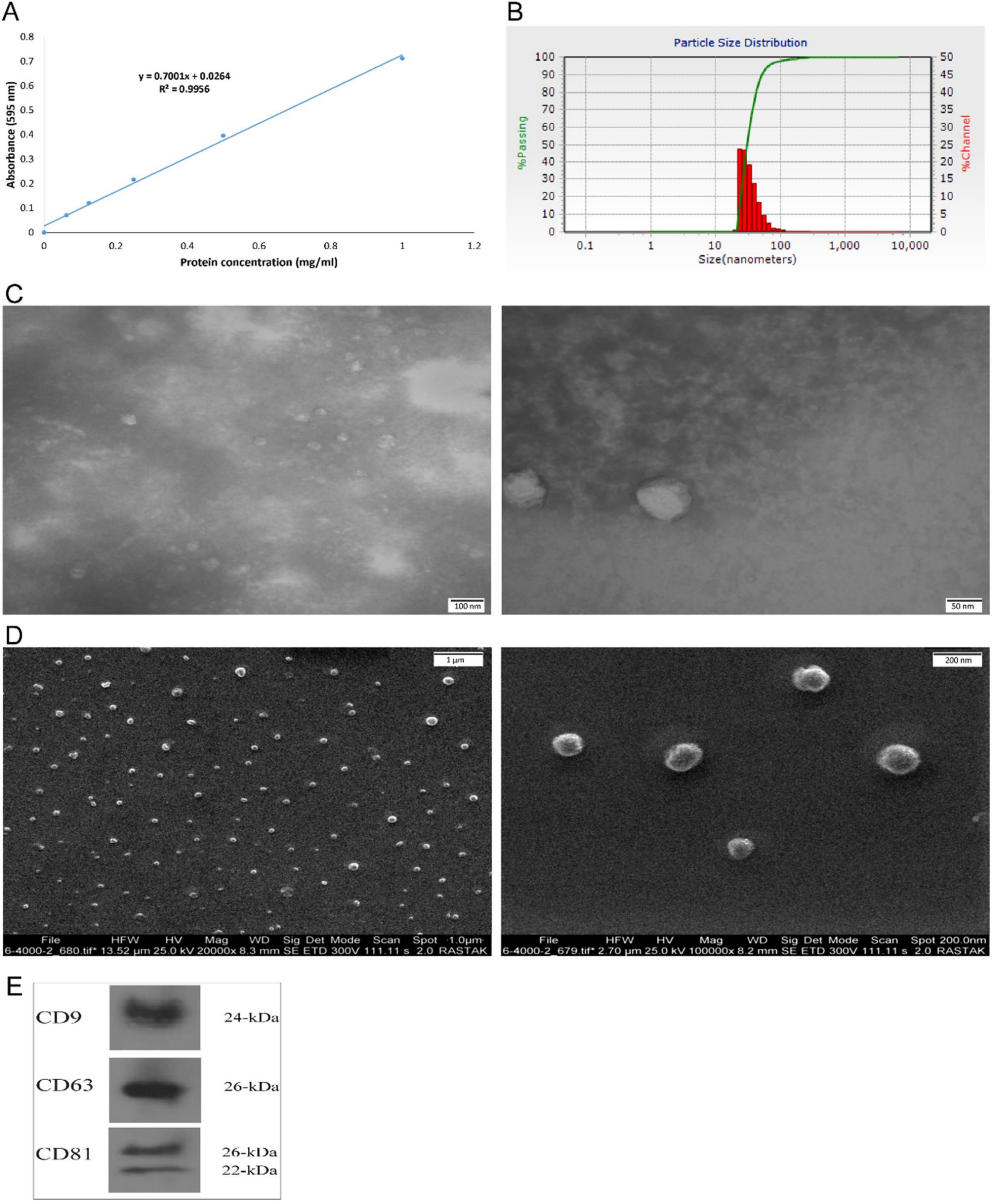
Characterization of osteoblast-like cell-derived exosomes: (**A**) Determination of exosome concentration using a standard curve based on the Bradford assay. (**B**) Measurement of exosome particle size distribution by DLS. (**C**, **D**) Morphological characterization of exosomes using Transmission Electron Microscopy (TEM) and FEI Environmental Scanning Electron Microscopy (ESEM). (**E**) Western blot analysis of exosomal surface markers

The ODExo were characterized using Bradford assay, DLS, TEM, SEM, and western blot analysis. The Bradford assay, along with its standard curve, indicated that the concentration of the isolated exosomes was approximately 1.2 mg/mL (Fig. 4A). As shown in Fig. 4B, DLS analysis revealed that the exosome size distribution predominantly fell within the range of 25 to 144 nm. TEM analysis demonstrated that most of the exosomes exhibited a characteristic cup-shaped morphology, with a central depression, and were within the average size range of 40 nm (Fig. 4C). SEM further confirmed the quality of the isolated exosomes, revealing a spherical shape with sizes ranging from 30 to 150 nm (Fig. 4D), corroborating the findings from DLS analysis. Furthermore, western blot analysis confirmed the presence of characteristic exosomal surface markers, including CD9, CD63, and CD81 (Fig. 4E), verifying their exosomal identity. Collectively, these findings confirm the successful isolation and characterization of exosomes.

### Exosome dose optimization for osteogenesis

**Fig. 5:**
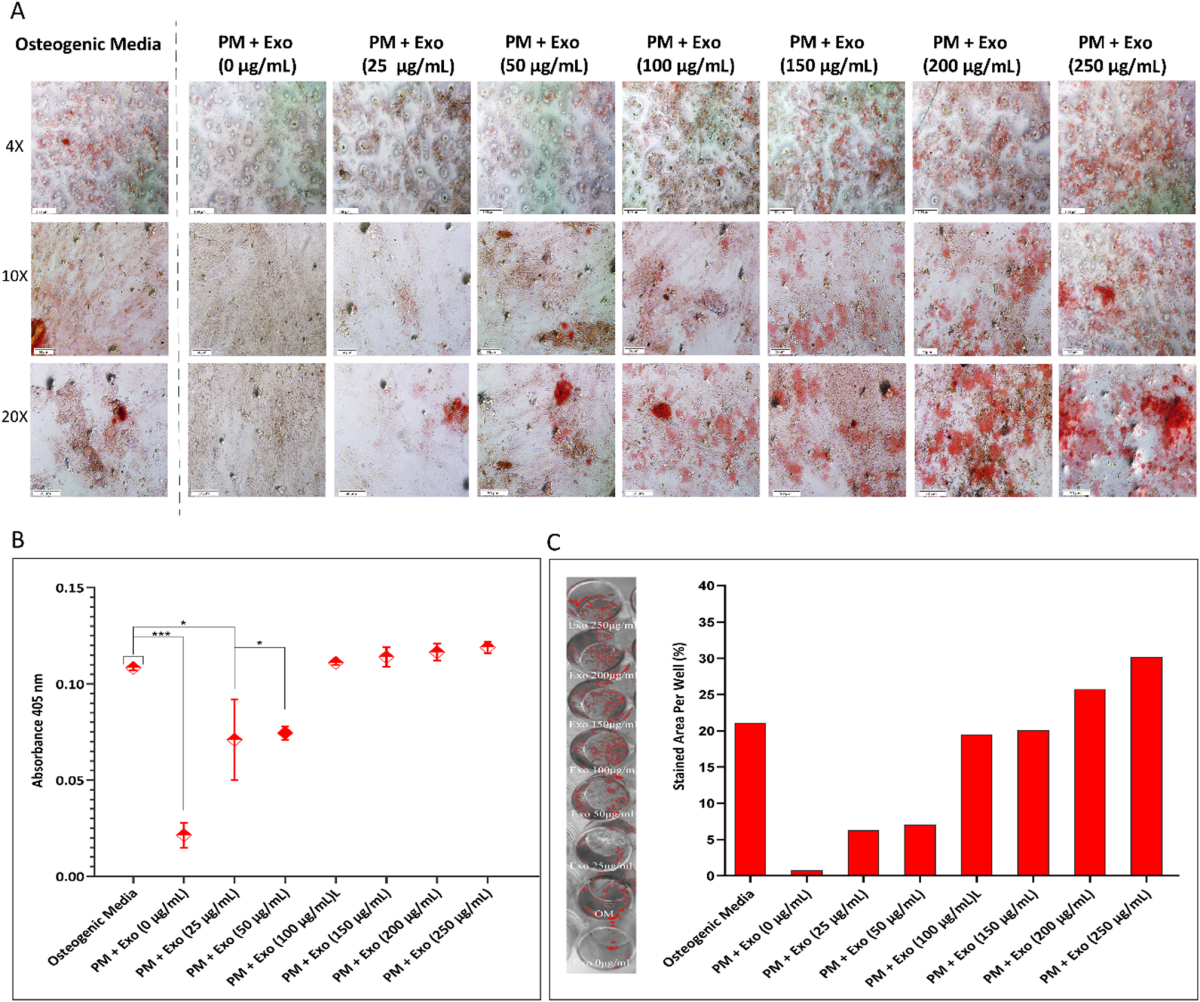
Determination of optimal exosome concentration for osteogenesis: (**A**) Representative microscopic images of calcium nodule formation in response to different exosome concentrations (0–250 µg/mL) and osteogenic medium (positive control) after 14 days. Increased mineralization was evident from 100 µg/mL onward. (**B**) Quantification of solubilized ARS dye (absorbance at 405 nm), showing significantly lower mineralization in the 0–50 µg/mL groups compared to the control, while no significant difference was observed in the 100–250 µg/mL groups. (**C**) Image J-based quantification of stained area supported the same trend. Data are presented as mean ± SD (*n* = 3). *p < 0.05; ***p < 0.001 vs. control group. Statistical analysis was performed using ordinary one-way ANOVA followed by multiple comparisons test

To evaluate the optimal concentration of ODExo for osteogenic induction, Alizarin Red S staining was employed as an indicator of calcium deposition (Fig. 5A). The presence of red mineralized nodules confirmed the osteoinductive effect of varying concentrations of exosomes compared to the osteogenic induction medium (positive control). Minimal calcium deposits were observed in the absence of Exo, while limited mineralization occurred at 25 and 50 µg/mL. In contrast, from 100 µg/mL onward, calcium deposition markedly increased, closely resembling the positive control. Notably, calcium deposition increased with exosome concentration up to 100 µg/mL, beyond which a significant further enhancement was not observed, suggesting a saturation or plateau effect likely due to limited cellular uptake or receptor saturation of osteogenic signaling pathways. These findings were further supported by quantitative analysis of the extracted Alizarin red staining dye (Fig. 5B), which showed a significant difference between the 0–50 µg/mL groups and the control, while no statistically significant difference was observed among the 100–250 µg/mL groups and the control, indicating comparable osteoinductive potential. Additionally, the stained area in each group was quantified using Image J software (Fig. 5C), which confirmed the trends observed in both qualitative and absorbance-based ARS analysis. Based on these findings, 100 µg/mL was selected as the optimal concentration for subsequent experiments.

### Real-time PCR analysis

**Fig. 6:**
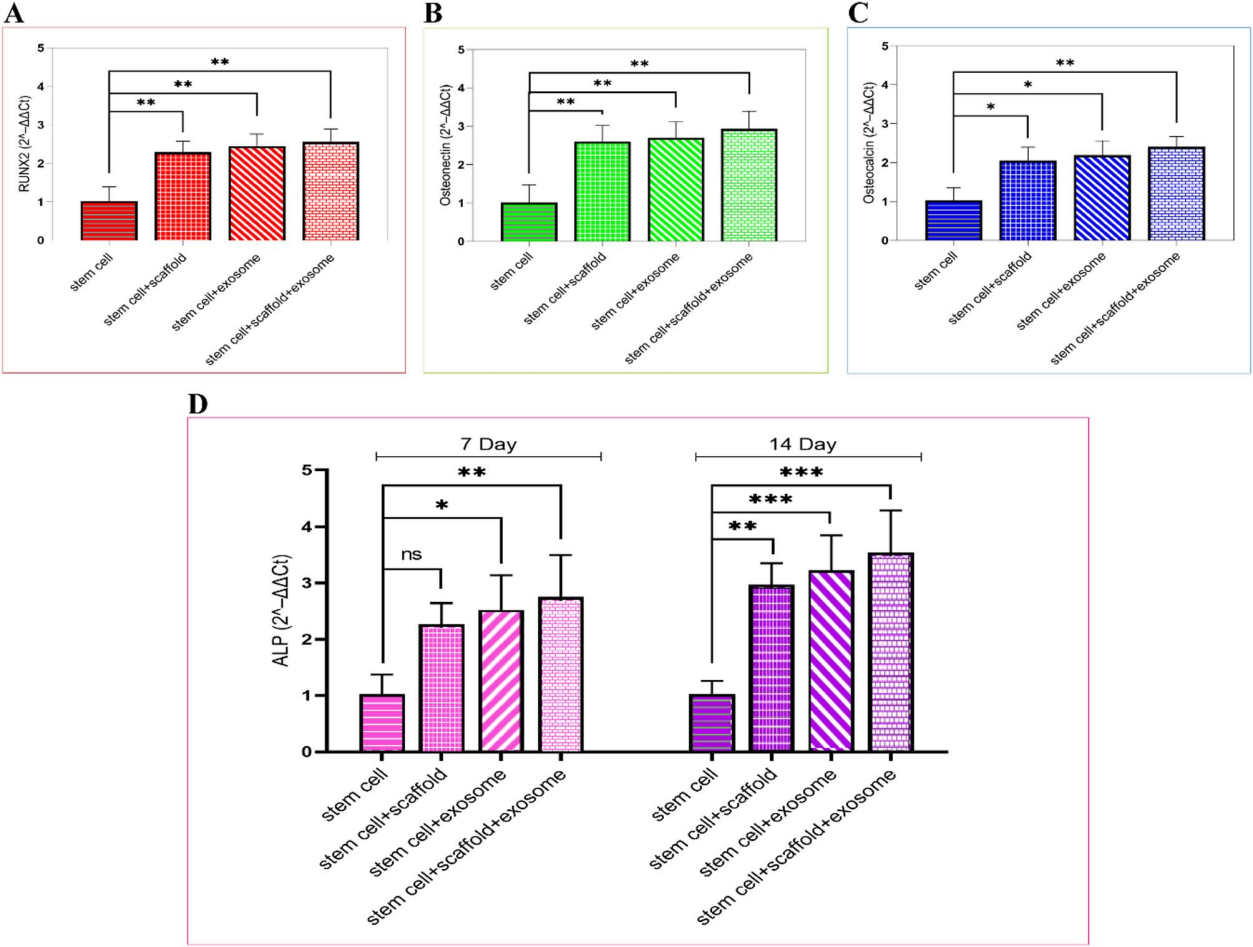
Quantitative real-time PCR: qRT-PCR analysis showing the relative expression levels of RUNX2 (**A**), Osteonectin (**B**), and Osteocalcin (**C**) at day 7, and ALP (**D**) at days 7 and 14 following treatment of hEnMSCs with PCL/Col scaffold, exosome, and the PCL/Col scaffold–exosome combination. Data are presented as mean ± SD (*P < 0.05, **P < 0.01, ***P < 0.001)

To evaluate the molecular characteristics of osteogenic differentiation in hEnMSCs treated with collagen-coated PCL scaffold, exosomes, or their combination, qRT-PCR was used to assess the mRNA expression levels of RUNX2, osteonectin, osteocalcin, and ALP relative to the untreated control group. According to Fig. 6A, on day 7, RUNX2 expression was significantly upregulated in all treated groups compared to the control (*P* < 0.01), emphasizing its central role in the initiation of osteogenesis. Osteonectin expression followed a similar trend, with all experimental conditions showing significantly elevated levels relative to the control (*P* < 0.01; Fig. 6B). In contrast, osteocalcin, a late-stage osteogenic marker involved in matrix mineralization, was expressed at comparatively lower levels across all groups on day 7 (Fig. 6C). However, its expression was significantly increased in the PCL/Col scaffold + exosomes group (*P* < 0.01), suggesting the onset of later-stage differentiation under combined treatment. As shown in Fig. 6D, ALP gene expression was markedly elevated following treatment with exosomes alone or in combination with scaffold. By day 14, ALP levels remained high or were further enhanced, particularly in the PCL/Col scaffold + exosomes group (*P* < 0.001), indicating sustained activation of osteogenic pathways beyond early induction stages.

### In vivo findings

#### Histological findings

##### Day 14

**Fig. 7:**
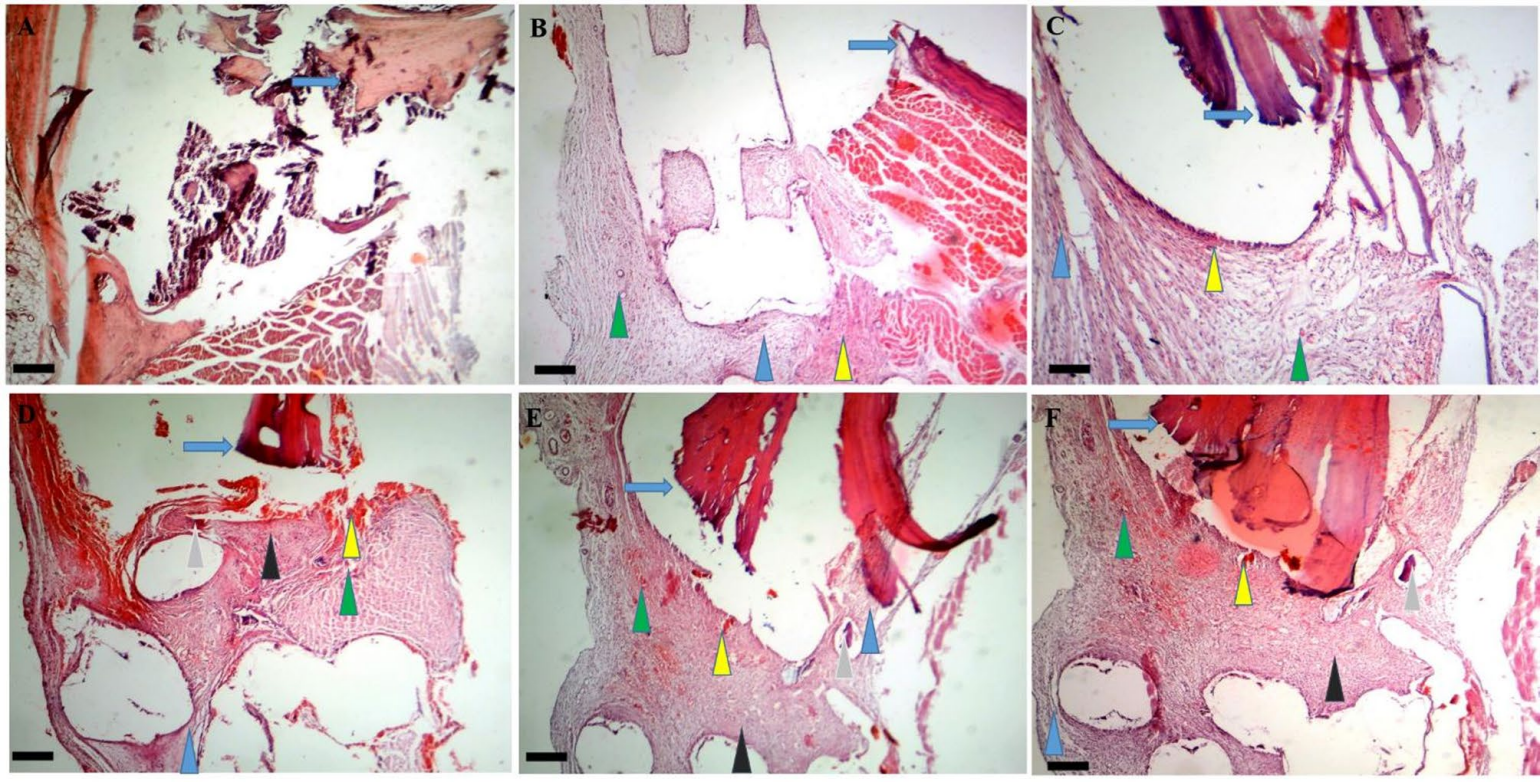
Histopathological bone scoring system in various groups on day 14 post-treatment: (**A**) in the control group fracture line (blue arrow) are observed. Images (**B** and **C**) represent the fracture line (blue arrow), fibrous tissue (blue arrowhead), vessels (green arrowhead) and new bone formation (yellow arrowhead) in the Exo and hEnMSCs groups. Images (**D**, **E**, and **F**) show the fracture line (blue arrow), fibrous tissue (blue arrowhead), vessels (green arrowhead), cartilage (black arrowhead), remaining scaffold (gray arrowhead) and new bone formation or osteoid (yellow arrowhead) in the PCL/Col, PCL/Col/Exo, and PCL/Col/Exo/hEnMSCs groups, respectively. Scale bar: 56 μm

On day 14 post-treatment, the mean bone regeneration score (BRS) were calculated as 2.33 ± 0.57 for the control, 5.66 ± 0.58 for the Exo, 6.33 ± 1.15 for the hEnMSCs, 7 ± 1 for the PCL/Col, 9.33 ± 0.56 for the PCL/Col/Exo, and 12.0 ± 1.0 for the PCL/Col/Exo/hEnMSCs groups. All treatment groups showed the higher BRS than that of the control groups (*p* < 0.05). The PCL/Col/Exo and PCL/Col/Exo/hEnMSCs groups showed significantly higher BRS than those of the Exo (*p* = 0.006, *p* = 0.025, respectively), hEnMSCs group (*p* < 0.001, *p* = 0.002, respectively). The PCL/Col/Exo/hEnMSCs group showed significantly higher BRS than that of the PCL/Col (*p* < 0.001) and PCL/Col/Exo groups (*p* = 0.002). The histopathological images are shown in Fig. 7.

##### Day 28

**Fig. 8:**
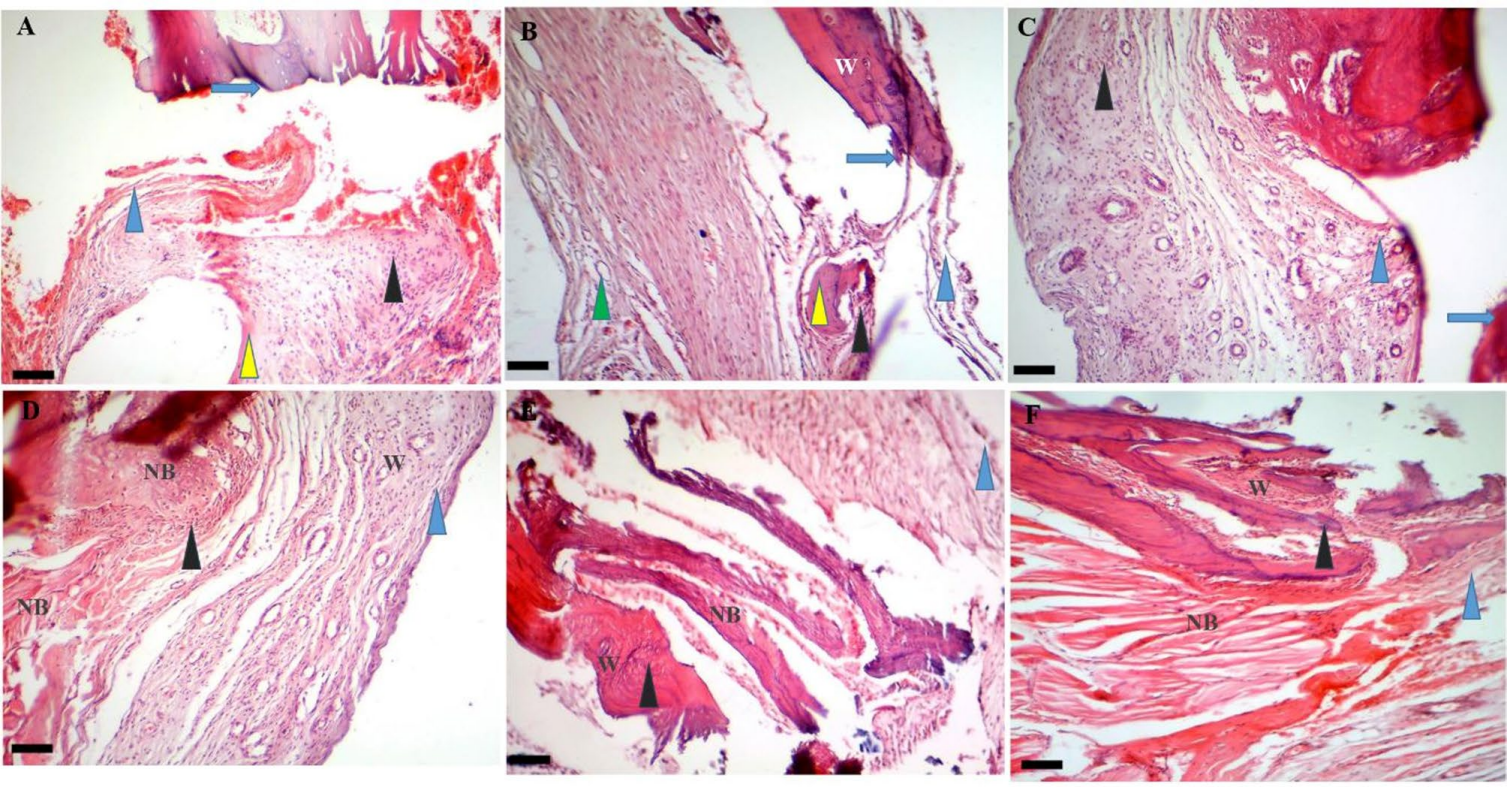
Histopathological bone scoring system in various groups on day 28 post-treatment: Fracture line (blue arrow), fibrous tissue (blue arrowhead), vessels (green arrowhead), cartilage (black arrowhead), and new bone formation (yellow arrowhead) are observed in the control, Exo, and hEnMSCs groups (**A** to **C**, respectively). Images (**D**, **E**, and **F**) show the fracture line (blue arrow), fibrous tissue (blue arrowhead), cartilage (black arrowhead) and new bone (NB) formation and Woven bone (W) in the PCL/Col, PCL/Col/Exo, and PCL/Col/Exo / hEnMSCs groups, respectively. Scale bar: 28 μm

On day 28 post-treatment, the mean BRS was calculated as 12.34 ± 1.15 for the control group, 13.00 ± 1.73 for the Exo, 13.66 ± 0.58 for the hEnMSCs, 16.00 ± 2.00 for the PCL/Col and 19.66 ± 1.52 for the PCL/Col/Exo and 22.0 ± 2.64 for the PCL/Col/Exo/hEnMSCs groups. The PCL/Col/Exo and PCL/Col/Exo/hEnMSCs groups showed significantly higher BRS than all other groups (*p* < 0.05). There was no significant difference between other groups in terms of BRS at this time (*p* > 0.05). The histopathological images are shown in Fig. 8.

##### Day 42

**Fig. 9:**
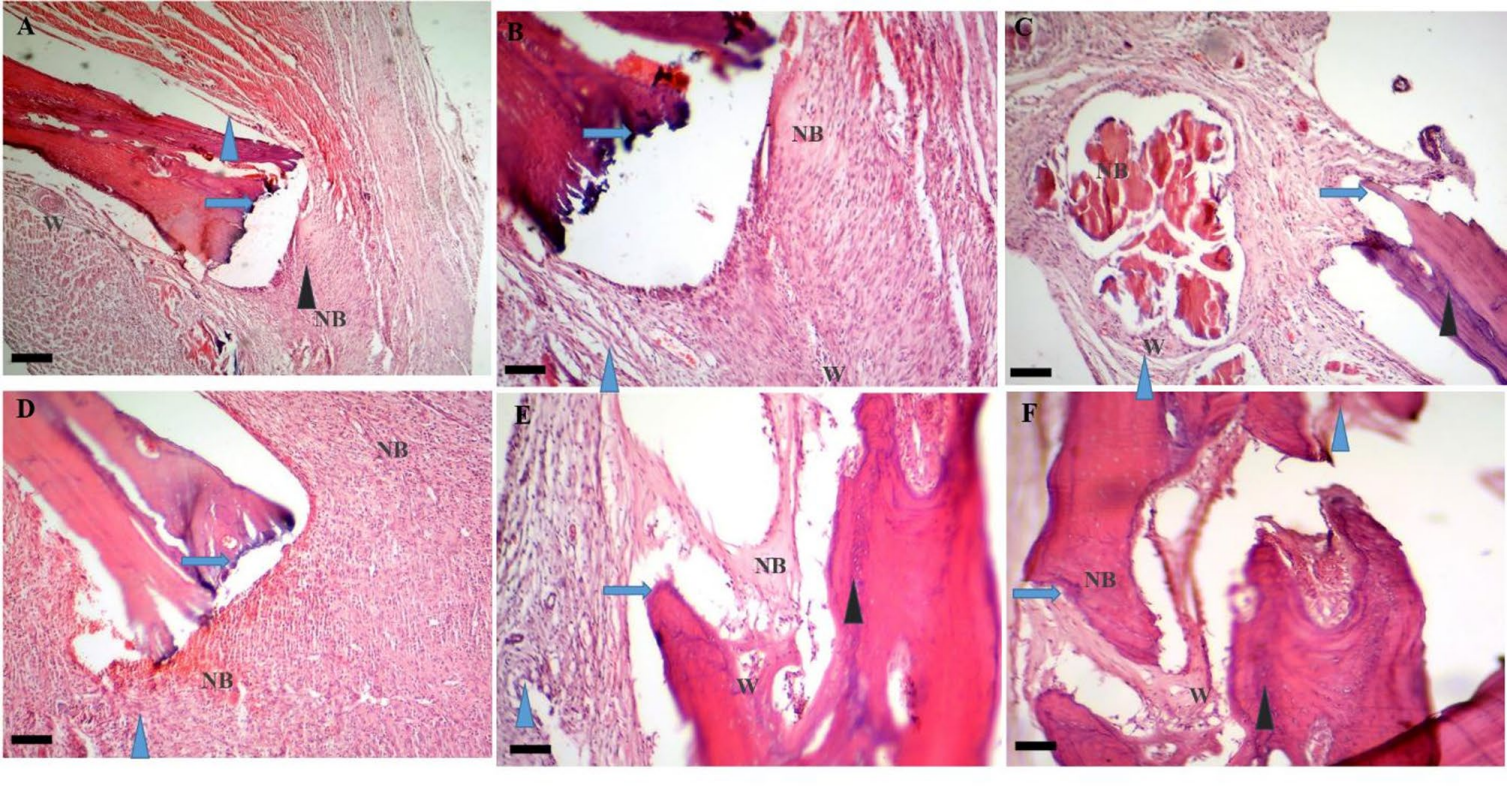
Histopathological bone scoring system in various groups on day 42 post-treatment: Fracture line (blue arrow), fibrous tissue (blue arrowhead), cartilage (black arrowhead), and new bone formation (NB), and Woven bone (W) are observed in the control, Exo, and hEnMSCs groups (**A** to **C**, respectively). Images (**D**, **E**, and **F**) show the fracture line (blue arrow), fibrous tissue (blue arrowhead), cartilage (black arrowhead) and new bone (NB) formation and Woven bone (W) in the PCL/Col, PCL/Col/Exo, and PCL/Col/Exo / hEnMSCs groups, respectively. Scale bar: 28 μm

On day 42 post-treatment, the mean BRS was calculated as 17.31 ± 1.52 for the control, 18.3 ± 1.51 for the Exo, 16.60 ± 2.06 for the hEnMSCs, 23.0 ± 1.0 for the PCL/Col, 27.33 ± 1.53 for the PCL/Col/Exo, and 29.0 ± 2.0 for the PCL/Col/Exo/hEnMSCs groups. The PCL/Col/Exo/hEnMSCs, PCL/Col/Exo and PCL/Col groups showed significantly higher BRS than Exo and hEnMSCs groups (*p* < 0.05). The PCL/Col/Exo/hEnMSCs and PCL/Col/Exo groups also showed significantly higher BRS than that of the PCL/Col group (*p* = 0.05 and *p* = 0.008, respectively). The histopathological images are shown in Fig. 9.

##### Day 56

**Fig. 10:**
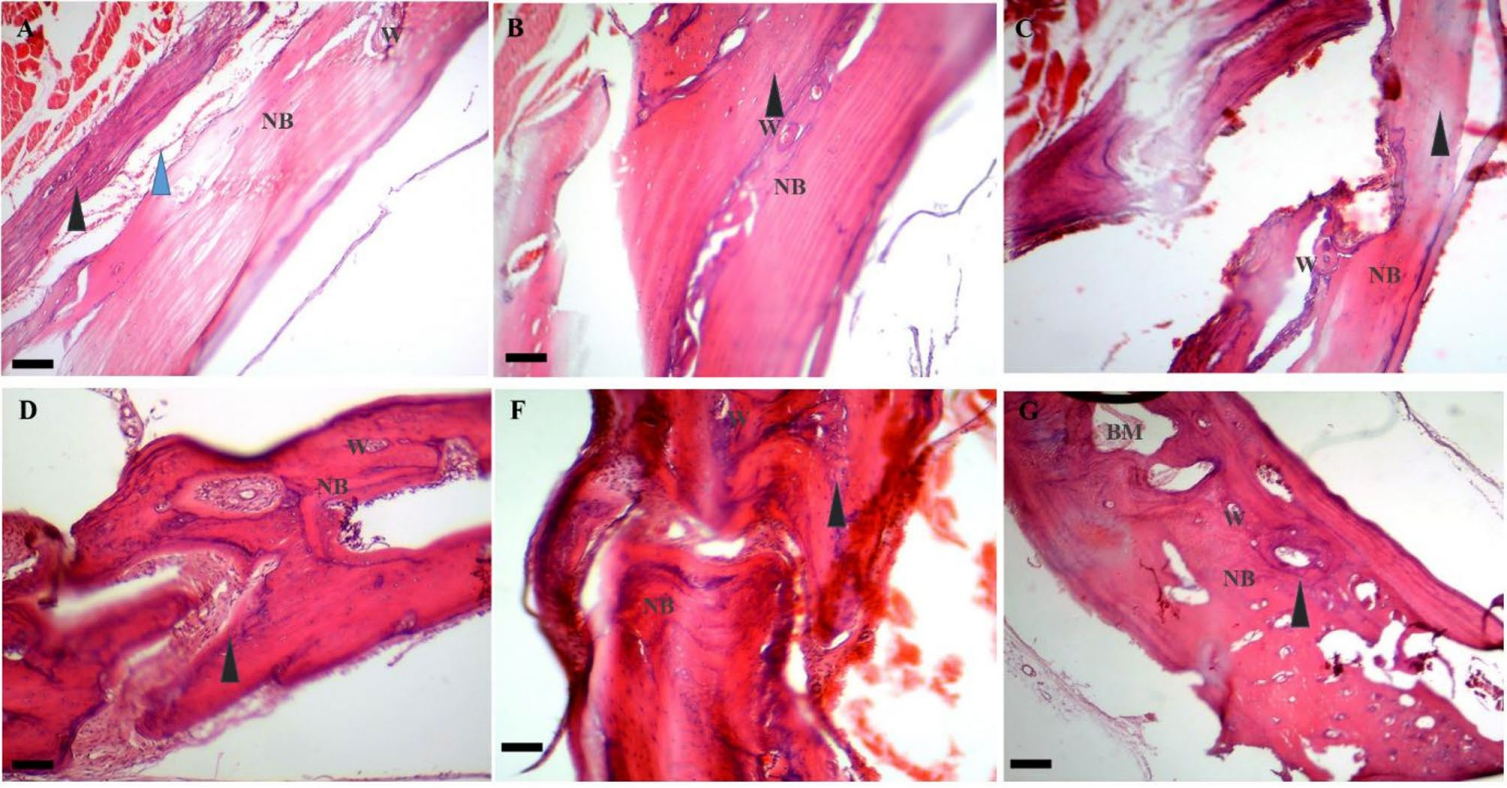
Histopathological bone scoring system in various groups on day 56 post-treatment: fibrous tissue (blue arrowhead), cartilage (black arrowhead), and new bone formation (NB), and Woven bone (W) are observed in the control, Ex, and hEnMSCs groups (**A** to **C**, respectively). Images (**D** and **E**) show fibrous tissue cartilage (black arrowhead) and new bone (NB) formation and Woven bone (W) in the PCL/Col, PCL/Col/Exo, groups, respectively. Image F shows cartilage (black arrowhead), bone marrow (BM) and new bone (NB) formation and Woven bone (W) in the PCL/Col/Exo / hEnMSCs group. Scale bar: 28 μm

On day 56 post-treatment, the mean BRS was calculated as 23.0 ± 1.73 for the control, 24.00 ± 2.64 for the Exo, 25.3 ± 1.51 for the hEnMSCs, 27.66 ± 3.05 for the PCL/Col, 32.33 ± 1.51 for the PCL/Col/Exo, and 34.66 ± 2.5 for the PCL/Col/Exo/hEnMSCs groups. The PCL/Col/Exo/hEnMSCs group showed significantly higher BRS than all other groups (*p* < 0.05). Except the PCL/Col, the PCL/Col/Exo group showed significantly higher BRS than all other groups (*p* < 0.05). There was no significant difference between other groups in terms of BRS at this time (*p* = 0.030). The histopathological images are shown in Fig. 10.

## Discussion

The simultaneous use of cell-free, 3D printed scaffolds functionalized with biological agents such as osteoblast-derived exosomes has emerged as a novel and promising approach in BTE. While previous studies have demonstrated that polycaprolactone, collagen, and exosomes individually contribute to osteogenic differentiation, this study explored their combined effect within a unified scaffold system. For the first time, a porous 3D-printed PCL scaffold coated with Col and osteoblast-derived exosomes was employed to interact with hEnMSCs. Remarkably, this engineered construct was capable of inducing osteogenic differentiation in vitro, even in the absence of osteoinductive supplements typically required for bone lineage commitment, and significantly enhanced bone regeneration in an in vivo rat model.

Extrusion-based 3D printing has emerged as a widely adopted technique for the fabrication of BTE scaffolds due to its ability to produce complex and patient-specific geometries with high resolution, reproducibility, and cost efficiency. This method offers precise control over scaffold architecture, enabling the fabrication of structures with interconnected porosity and mechanical properties suitable for osteogenic applications [15, 18, 43]. Polycaprolactone is a widely used FDA-approved thermoplastic synthetic polymer with excellent printability, enabling the fabrication of scaffolds with defined porosity and precise layer-by-layer geometry through 3D printing technologies [44–46]. Numerous studies have demonstrated that scaffold pore sizes in the range of 100 to 400 μm are optimal for supporting cell infiltration, migration, and proliferation in BTE applications [47, 48]. In the present work, the fabricated PCL scaffolds exhibited a regular and uniform architecture, with an average pore size of approximately 300 μm and a strand diameter of around 500 μm. Notably, Cakmak, Unal [49] and Wang, Gu [50] reported that pore sizes near 300 μm are particularly favorable for bone ingrowth and effective bone tissue regeneration.

In addition, PCL is considered a desirable material for bone scaffold fabrication due to its controlled biodegradability, high biocompatibility, favorable mechanical properties, and excellent surface modifiability [18, 20, 21]. In the present study, Col coating were applied to the surface of the PCL scaffolds to improve hydrophilicity, enhance cell adhesion, and overcome the inherent lack of osteoinductivity of pristine PCL. These findings are supported by previous studies which demonstrated that Col functionalization significantly enhances the hydrophilicity and cellular adhesion of PCL scaffolds, while also promoting osteogenic differentiation and improving biocompatibility [23, 51]. FTIR findings also confirmed the successful incorporation of Col onto the scaffold surface. Specifically, distinct absorption peaks corresponding to amide I (~ 1655 cm⁻¹) and amide II (~ 1551 cm⁻¹), characteristic of Col, were observed in the coated samples, but were absent in uncoated PCL. Furthermore, a broad band near 3325 cm⁻¹, attributed to N–H stretching (amide A), indicated the presence of hydrogen bonding within the Col network.

In tissue engineering, scaffolds act as temporary matrixes that support the replacement of the newly regenerated tissue with scaffold material, provided the scaffold’s degradation rate is appropriately aligned with the regeneration rate of the target tissue [19]. Therefore, a degradation test was conducted to evaluate the structural stability of Col-coated 3D-printed scaffolds under simulated physiological conditions designed for bone tissue regeneration. Porosity, swelling behavior, and degradation rate are critical physicochemical parameters that significantly influence the functional performance of scaffolds in BTE. In the present study, the Col-coated PCL scaffolds exhibited a porosity of approximately 54%, which lies within the optimal range of 40–90% as reported in previous studies [52, 53]. This porosity has been shown to provide a favorable balance between mechanical integrity and biological activity, supporting both structural stability and tissue regeneration. Specifically, such porosity enables sufficient space for cellular infiltration, proliferation, and nutrient exchange, while preserving the mechanical strength required for load-bearing applications [54].

The swelling behavior of scaffolds plays a critical role in modulating both mechanical integrity and biological functionality by promoting cell adhesion and protein adsorption [55]. In this study, the swelling ratio reached approximately 6.14% after 24 h, suggesting an increase in hydrophilicity attributed to the collagen coating. This result is consistent with the findings of Ebrahimi, Irani [23], who also reported that the incorporation of collagen led to a noticeable improvement in the water absorption capacity of PCL scaffolds, which are inherently hydrophobic in nature.

Furthermore, the scaffolds exhibited a gradual and controlled degradation profile, with a total weight loss of approximately 10.74% over 60 days. This degradation behavior is well-aligned with the expected time course of new bone formation and is comparable to previous studies using PCL-based systems (e.g.,Ebrahimi, Irani [23]).

The results of the compression test revealed that the fabricated scaffold exhibited a compressive modulus of 58.4 MPa, which falls within the range reported for trabecular and low-density cortical bone [56]. While this may suffice for moderate-load applications, higher mechanical demands, such as in high-density cortical bone, may require reinforcement. Although no additives were used in this study, previous reports have shown that incorporating hydroxyapatite (HAp) into PCL scaffolds can significantly improve compressive properties [57, 58].

Biocompatibility is recognized as a fundamental prerequisite for scaffolds used in BTE, as it directly influences the material’s ability to support cellular attachment, viability, proliferation, and differentiation without eliciting adverse immune responses [59]. In the context of polymer-based scaffolds such as PCL, enhancing surface characteristics, particularly by improving hydrophilicity and introducing bioactive cues, has become a widely adopted strategy to promote scaffold biocompatibility and facilitate effective cell–scaffold interactions. In this regard, Col coating of PCL scaffolds has been shown to enhance the attachment and proliferation of three different cell types under in vitro conditions [19].

Based on these findings, and in order to further assess the biocompatibility of the scaffolds developed in the present study, MTT assay, DAPI staining, and SEM imaging were employed to evaluate cell adhesion, viability, and morphological spreading on the 3D-printed constructs. The MTT assay revealed a time-dependent increase in hEnMSC viability on both the Col-coated PCL scaffolds and the TCP control, particularly on days 3 and 5. This result indicates that the scaffolds provided a metabolically supportive microenvironment without inducing cytotoxic effects. These findings are in agreement with a previous report in which enhanced cell proliferation on Col-coated 3D-printed PCL scaffolds has been shown [60], further supporting the role of Col in improving scaffold biocompatibility and promoting cellular metabolic activity in vitro in support of the MTT findings, DAPI staining further confirmed the biocompatibility and pro-proliferative nature of the Col-coated PCL scaffolds. Nuclei staining at days 1 and 3 revealed a progressive increase in cell density, with hEnMSCs uniformly distributed across the scaffold surface. According to various studies, this homogeneous pattern indicates successful initial cell attachment and propagation is stable during the initial cultivation period [19, 23].

Further insights into cell–scaffold interactions were obtained through SEM imaging after 3 days of culture, demonstrating clear evidence of cell adhesion and morphological integration, with cells displaying flattened morphologies and extended contact areas, observable from both top and side views. These ultrastructural features underscore the scaffold’s ability to provide a favorable microenvironment for cell anchorage, spreading, and cytoskeletal engagement—hallmarks of early osteogenic compatibility.

Exosomes are small EVs containing RNA, proteins, and bioactive lipids that have been proposed in recent years as key paracrine mediators in tissue regeneration, particularly in bone repair [61, 62].

In addition to the well-established role of stem cell-derived exosomes, emerging evidence highlights the significance of exosomes derived from alternative cellular sources, particularly osteoblast-like cells, as recognized models for exploring osteogenic signaling pathways and assessing the differentiation potential of stem cells. The incorporation of SAOS-2-derived exosome into 3D-printed magnetic PLA scaffolds as a promising strategy for BTE applications has been previously highlighted. Their study demonstrated that these exosome-loaded constructs significantly enhance osteogenic differentiation and mineralization, underscoring their potential in promoting effective bone regeneration.

In the present study, exosomes were isolated from the conditioned medium of an osteoblast-like cell line commonly used in bone regeneration studies due to its robust osteoinductive capacity and stable osteogenic phenotype [63]. The successful isolation and identification of small EVs in our study were confirmed using standard biophysical and morphological techniques. The DLS analysis revealed a particle size distribution predominantly between 25 and 144 nm, consistent with the established size range for exosomes. Furthermore, TEM showed the classical cup-shaped morphology and a mean particle diameter of approximately 40 nm, while SEM imaging confirmed spherical vesicles with a comparable size range (30–150 nm), indicating the structural integrity and homogeneity of the isolated vesicles. Western blotting further supported the exosomal identity by detecting canonical markers such as CD9, CD63, and CD81 [63, 64]. Based on these validated features, the isolated exosomes were considered suitable for downstream applications in scaffold-mediated BTE.

The osteogenic induction efficacy of exosome-based strategies in bone tissue engineering is highly dose-dependent, as exosome concentration directly influences cellular uptake, signaling activation, and downstream osteogenic responses. Several studies have emphasized that low concentrations may be insufficient to stimulate osteogenesis, while excessively high doses may lead to saturation or off-target effects [65, 66]. Exosomes derived from osteogenically induced hASCs have been shown to exhibit the strongest mineralization capacity at an intermediate dose, surpassing both untreated controls and standard osteogenic medium. Similarly, another study evaluating exosomes derived from human umbilical cord MSCs tested concentrations of 0, 100, and 200 µg/mL, and found that 100 µg/mL resulted in the most significant enhancement of osteogenic differentiation, as demonstrated by increased calcium deposition and Alizarin Red S staining [67].

In our study, exosome concentration was standardized using total protein content quantified by the Bradford assay that is a common surrogate for particle number in in vitro experiments. A basal protein concentration of approximately 1.2 mg/mL was used to prepare serial dilutions for cellular assays. Alizarin Red S staining revealed a clear dose-dependent increase in calcium deposition, with minimal mineralization at concentrations ≤ 50 µg/mL. In contrast, 100 µg/mL and higher induced robust nodule formation, comparable to that observed with osteogenic induction medium. Quantitative ARS analysis also confirmed that 100 µg/mL represented the optimal threshold for inducing a strong osteogenic response in hEnMSCs, compared to both the Exo-free group and the group treated with osteogenic medium alone. Interestingly, SAOS-2–derived exosomes, have recently been reported to be effective osteogenic outcomes at a dose of 50 µg/mL when the vesicles were incorporated into magnetic PLA scaffolds, which is inconsistent with our study. Although this concentration was lower than the optimal threshold identified in our findings, the results further highlight the osteoinductive potential of osteoblast-derived exosomes across distinct delivery systems. While the authors did not explicitly link the dose-dependent efficacy to scaffold properties, the use of magnetic PLA may have promoted enhanced exosome retention or localized release, potentially enabling robust biological effects at reduced concentrations [16].

Quantitative gene expression analysis in our study further validated the osteoinductive potential of exosomes derived from human osteoblast-like cells at a concentration of 100 µg/mL in hEnMSCs. Among the early osteogenic markers, ALP expression was significantly upregulated on day 7 in both the exosome-treated and PCL/Col scaffold + exosomes groups compared to the cell-only control, with the highest level observed in the combined condition (*p* < 0.001). This elevation was further amplified by day 14, particularly in the PCL/Col scaffold + exosome group, which maintained the most robust expression profile. These findings are consistent with previous studies identifying ALP as a reliable early marker of osteogenic differentiation, and sustained expression indicates continued osteoblastic activity, likely supported by the bioactive and biomimetic nature of the exosome-loaded scaffold [16, 65, 68]. Notably, only minor differences in ALP gene expression were observed between the exosomes and PCL/Col scaffold + exosomes groups at both 7 and 14 days, suggesting that the presence of the 3D scaffold did not markedly enhance early transcriptional activity beyond that induced by exosome treatment alone. Several plausible explanations may account for this finding. Exosomes inherently contain a potent cargo of osteoinductive biomolecules, such as miRNAs, proteins, and growth factors, that can independently activate robust osteogenic differentiation even without additional 3D cues. Once these signaling pathways are strongly engaged, the contribution of scaffold-derived mechanical or spatial cues may become less pronounced, leading to a ceiling or saturation effect in gene expression. Moreover, the evaluated time points (7 and 14 days) primarily correspond to early differentiation phases, when scaffold-mediated mechanical and architectural effects on matrix formation are not yet fully manifested. In addition, the structural characteristics of the collagen-coated PCL scaffold optimized mainly to promote cell adhesion and 3D organization may not substantially amplify short-term transcriptional activity. The diffusion and release kinetics of exosomes within the porous structure could also result in similar local exosome availability between conditions, further minimizing expression differences. Taken together, these factors suggest that the comparable ALP expression reflects biological saturation and temporal dynamics of osteogenic signaling, rather than limitations in scaffold design or experimental setup.

Elevated RUNX2 expression across all treatment groups further underscores its pivotal role in osteoblast lineage specification. As a master transcription factor, RUNX2 governs the early commitment of MSCs to the osteogenic pathway. Notably, the highest expression levels were again observed in the PCL/Col scaffold + exosomes group, suggesting a synergistic interplay between exosomal bioactive cargo and the 3D scaffold matrix, which may enhance transcriptional activation of osteogenic genes [69]. Similarly, osteonectin expression remained consistently upregulated, particularly in PCL/Col scaffold + exosomes conditions, supporting its function in collagen binding and calcium regulation during early extracellular matrix organization. In contrast, osteocalcin, a late-stage marker of matrix maturation, was less expressed overall, consistent with its temporal placement in the osteogenic cascade. Nevertheless, its significant elevation in the PCL/Col scaffold + exosomes group suggests that this combinatory system not only initiates osteogenesis but also facilitates its progression through to matrix mineralization [16, 69]. Taken together, the concurrent upregulation of both early and late markers in the PCL/Col scaffold + exosomes group reinforces the hypothesis that exosome-based signaling, when delivered within a 3D scaffold, creates a biomimetic niche capable of supporting full-phase osteogenic differentiation in hEnMSCs.

The ability of synthesized scaffolds to osteogenesis induction was evaluated finally in a rat model of bone calvarial defect at four time intervals as previously reported by Bakhtiarimoghadam, Shirian [41]. The data obtained from the histological sections showed that the synthesized scaffolds were degraded four weeks after treatment. The highest BRS was detected in the PCL/Col/Exo/hEnMSCs group followed by PCL/Col/Exo at any four-time interval. Firstly, these findings show that the synthesized scaffold, hEnMSCs, and ODExo alone cannot accelerate bone regeneration. Secondly, the higher BRS in the PCL/Col/Exo/hEnMSCs than that of the PCL/Col/Exo indicates that osteoblast-derived exosomes could enhance hEnMSCs-induced osteogenesis when they are seeded on a 3D printed PCL/Col. It has been recently shown that transplantation of PCL/bone marrow MSCs/self-assembling peptides composite implants clearly promoted bone regeneration and neovascularization [70]. In addition, small EVs from 3D PCL/hydroxyapatite scaffold promote bone regeneration and vascular formation [71]. The osteogenic potential of skeletal muscle-derived EVs has been shown to promote osteogenic differentiation of MSCs but also protect against disuse osteoporosis by enhancing glycolysis [72].

## Conclusion

The synthesized 3D printed PCL/Col scaffolds demonstrated suitable physicochemical properties, controlled porosity, mechanical integrity, and improved surface hydrophilicity, which collectively enhanced cell adhesion, proliferation, and osteogenic differentiation in vitro, as evidenced by up-regulation of ALP, OCN, RUNX2, and osteonectin. Transplantation of PCL/Col scaffolds seeded with human endometrial mesenchymal stem cells (hEnMSCs) and osteoblast-derived exosomes further promoted bone regeneration in a rat calvarial defect model, accelerating matrix mineralization and demonstrating the synergistic effect of cellular and exosomal components. These findings highlight the potential of 3D printed PCL/Col scaffolds combined with bioactive components as a promising strategy for bone tissue engineering, offering both cell-based and cell-free regenerative approaches. Despite these encouraging results, more comprehensive studies are needed to evaluate the long-term efficacy and potential clinical applications of these scaffolds.

## Supplementary Information

Below is the link to the electronic supplementary material.

Supplementary Material 1

## Data Availability

Data supporting the results presented in this article can be obtained from the corresponding author upon reasonable request.

## Abbreviations

BTE: Bone tissue engineering
PCL: Polycaprolactone
Col: Collagen
hEnMSCs: Human Endometrial Mesenchymal Stem Cells
ODExo: Osteoblast-Derived Exosomes
ECM: Extracellular matrix
Exo: Exosomes
qRT-PCR: Quantitative Real-time PCR
ARS: Alizarin Red S
ALP: Alkaline phosphatase
OCN: Osteocalcin
RUNX2: Runt-related transcription factor 2
ON: Osteonectin
