# Supplementary figures and images for: Exosome-functionalized collagen-coated 3D-printed PCL scaffold for enhanced osteogenic differentiation and bone regeneration: an in vitro and in vivo study
Source: J Biol Eng. 2025 Nov 27;19:107. doi: 10.1186/s13036-025-00578-w (PMC12661825; doi:10.1186/s13036-025-00578-w)

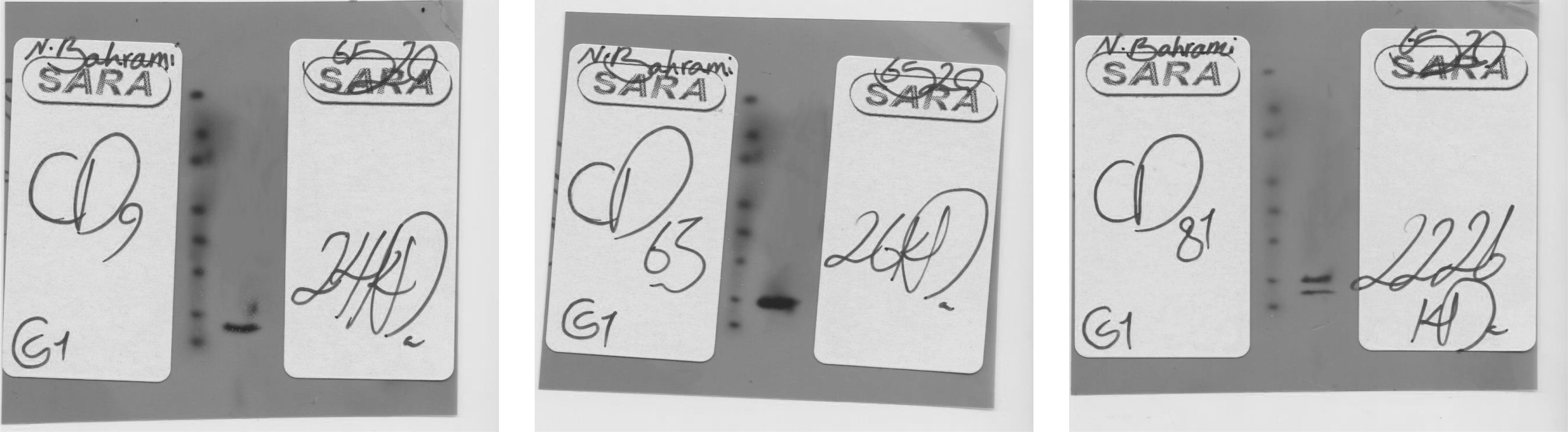

Supplement: Supplementary file 1 — Supplementary Material 1 [file 13036_2025_578_MOESM1_ESM.tif]
